# Anatomical and subcortical invasiveness in diffuse low-grade astrocytomas differ between IDH status and provide prognostic information

**DOI:** 10.48101/ujms.v129.10799

**Published:** 2024-09-03

**Authors:** Maria Zetterling, Markus Fahlström, Francesco Latini

**Affiliations:** a Department of Medical Sciences, Section of Neurosurgery, Uppsala University, Uppsala, Sweden; b Department of Surgical Sciences, Radiology, Uppsala University, Uppsala, Sweden

**Keywords:** Astrocytomas, low-grade gliomas, IDH status, DTI, white matter, Brain-Grid

## Abstract

**Background:**

Diffuse astrocytomas preferentially infiltrate eloquent areas affecting the outcome. A preoperative understanding of isocitrate dehydrogenase (IDH) status may offer opportunities for specific targeted therapies impacting treatment management. The aim of this study was to analyze clinical, topographical, radiological in WHO 2 astrocytomas with different IDH status and the long-term patient’s outcome.

**Methods:**

A series of confirmed WHO 2 astrocytoma patients (between 2005 and 2015) were retrospectively analyzed. MRI sequences (FLAIR) were used for tumor volume segmentation and to create a frequency map of their locations into the Montreal Neurological Institute (MNI) space. The Brain-Grid (BG) system (standardized radiological tool of intersected lines according to anatomical landmarks) was used as an overlay for infiltration analysis of each tumor. Long-term follow-up was used to perform a survival analysis.

**Results:**

Forty patients with confirmed IDH status (26 IDH-mutant, IDHm/14 IDH-wild type, IDHwt) according to WHO 2021 classification were included with a mean follow-up of 7.8 years. IDHm astrocytomas displayed a lower number of BG-voxels (*P* < 0.05) and were preferentially located in the anterior insular region. IDHwt group displayed a posterior insular and peritrigonal location. IDHwt group displayed a shorter OS compared with IDHm (*P* < 0.05), with the infiltration of 7 or more BG-voxels as an independent factor predicting a shorter OS.

**Conclusions:**

IDHm and IDHwt astrocytomas differed in preferential location, number of BG-voxels and OS at long follow-up time. The number of BG-voxels affected the OS in IDHwt was possibly reflecting higher tumor invasiveness. We encourage the systematic use of alternative observational tools, such as gradient maps and the Brain-Grid analysis, to better detect differences of tumor invasiveness in diffuse low-grade gliomas subtypes.

## Introduction

Gliomas are derived from glial cells and comprise approximately 30% of all primary central nervous system tumors and 80% of malignant brain tumors (1–3). Diffuse low-grade gliomas (DLGG) are World Health Organization (WHO5) grade 2 astrocytomas, characterized by slow growth but extensive infiltration. They occur mainly in adult life with a peak incidence around 30–40 years (4, 5). The clinical course of low-grade gliomas is diverse, but the majority of tumors tend to recur or transform into high-grade gliomas and will eventually lead to death. Molecular features such as isocitrate dehydrogenase (IDH) mutation status and 1p19q codeletion are currently used in the classification of DLGG and applied in treatment-planning decisions (3). The systematic study on the molecular details of glioma cells level have an advanced understanding of the metabolic effects induced by IDH mutations, offering opportunities for specific targeted therapies that may improve patient outcomes (6). IDH-mutant WHO grade 2 astrocytomas are associated with a better overall survival (OS) than their IDH wild-type counterparts (7, 8).

These specific tumor features require an individualized approach for each patient to decide optimal treatment strategies (5, 9, 10). The extent of surgical resection is now established to be an important element affecting the overall survival independently of the molecular features (5, 6, 11–13). One of the major problems with the treatment strategy for DLGG is their tendency to grow in eloquent and not compensable areas, the so-called ‘minimal common brain’ (14–17). Among them, WHO 2 diffuse astrocytomas seem to have a tendency to infiltrate the white matter near the eloquent cortex or deep grey nuclei, making the risks of surgery higher (18–21).

Radiological features, the clinical situation and the neurosurgeon’s experience have been the crucial factors for the treatment decision-making at the moment of radiological diagnosis especially when awake surgery or advanced neuroimaging techniques such as magnetic resonance imaging (MRI)-based tractography were not commonly used. New individualized algorithms have been proposed to take into consideration radiology, tumor kinetics, and the patient-specific reorganization potential. The impact of infiltrated white matter, reflecting the host brain‑tumor interaction is therefore more relevant for the management at the individual level (9, 22). On the other hand, DLGGs are still classified by cortical nomenclature and their tumor volume computation (11, 13, 23, 24) underestimating their subcortical invasion and neglecting the possible biological and prognostic differences in tumors harboring within the same lobe (25). To integrate volume computation, topographical location, and subcortical extension, a radiological observational tool called the Brain-Grid was proposed (25). This technique seems to provide complementary information to the standard radiological topographical classification system for gliomas with more attention to their subcortical white matter extension (17, 25).

The objective of this study was to review the preferential location and subcortical extension of a retrospective cohort of diffuse low-grade astrocytoma patients with known IDH molecular status. We aimed to detect whether the different IDH status reflected topographical/radiological features, different clinical information, differences in the initial surgical management, and consequently on the outcome.

## Materials and methods

### Patient population

We collected clinical and radiological data for all patients with a radiological and histological diagnosis of WHO 2 astrocytomas between February 2005 and December 2015. All cases were reanalyzed to exclude cases of suspected higher-grade gliomas with contrast enhancement, poor quality of MRI (slice thickness > 3–5 mm), and high proliferation index (Ki67 > 10%). Molecular verification including the IDH status (either IDH1 or IDH2 mutant, IDHm; IDH wild type, IDHwt) and LOH1p19q codeletion were recollected in all the available cases according to the WHO 2021 classification (3). The regional ethics committee approved the study protocol (Dnr 2015-210-2, approval date was 2015-07-02). Data were collected retrospectively and anonymously as part of larger study; hence no informed consent from patients included in this study was needed.

### Neuroimaging, DTI acquisition and processing

**Figure 1:**
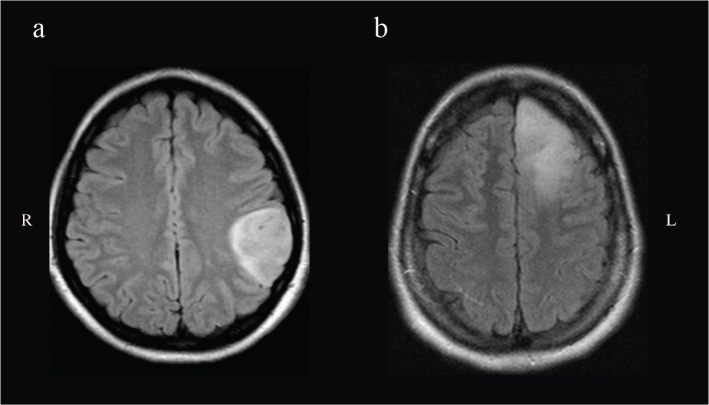
The picture shows axial FLAIR slices of two exemplary cases to demonstrate the difference in radiological tumor borders. In (a), a patient with astrocytoma harboring the left parietal lobe with well-defined tumor margins. In (b), the left frontal tumor displays irregular/diffuse margins in its posterior border.

Morphological MRI during the first 3 years of this retrospective study was performed on a Siemens Avanto 1.5 Tesla system (Siemens Healthcare, Erlangen, Germany) using a 22-channel head coil. The MRI acquisition protocol included a high-resolution contrast-enhanced 3D T1-weighted image (repetition time = 25 ms, echo time = 4.6 ms, flip angle = 30, resolution 1 × 1 × 1 mm^3^, 175 slices) and 2D fluid attenuated inversion recovery (FLAIR) image (repetition time = 6,000 ms, echo time = 120 ms, inversion time = 2,000 ms, flip angle = 90, resolution 0.5 × 0.5 × 2.4 mm^3^, 46 slices). As of 2007, MRI was performed on a Philips Achieva 3.0 Tesla system (Philips Healthcare, Best, the Netherlands) using a 32-channel head coil. The MRI acquisition protocol included a high-resolution contrast-enhanced 3D T1-weighted image (repetition time = 8.1 ms, echo time = 3.7 ms, flip angle = 8, resolution 1 × 1 × 1 mm^3^, 170 slices) and 2D or 3D fluid-attenuated inversion recovery (FLAIR) image (2D: repetition time = 11,000 ms, echo time = 125 ms, inversion time = 2,800 ms, flip angle = 90, resolution 0.5 × 0.5 × 6 mm^3^, 25 slices, 3D: repetition time = 4,800 ms, echo time =269 ms, inversion time = 1,650 ms flip angle = 90, resolution 1 × 1 × 0.5 mm^3^, 320 slices). Images acquired before the first operative intervention were used for tumor volume segmentation and visual analysis of neuroradiological features. Picture archiving and communication system (Vue PACS, version 11.1.0, Carestream Health Inc., Rochester, NY, USA) was used to segment the lesions with the aid of a semiautomatic method (livewire algorithm) (25–27). The tumor margins were considered well defined when outlined with a clear difference in signal intensity compared with peritumoral tissue (without any finger-like hyperintense signals on T2 or FLAIR sequences, Figure 1a) while tumor margins with unclear, and irregular signal intensity on FLAIR sequences was considered diffuse (Figure 1b). The transformation matrix describing the transformation from patient-specific space to Montreal Neurological Institute (MNI) space was derived based on the FLAIR images; the acquired transformation matrix was then applied to the segmented tumor volumes. Statistical maps of tumor location frequency were obtained by computing for each voxel the cumulative number of observed lesions (14). The Brain-Grid system (standardized radiological tool of intersected lines according to chosen anatomical landmarks) (25) was used for radiological and white matter analysis of the tumor location frequency map into MNI space. The Inferior fronto-occipital fasciculus (IFOF), the arcuate fasciculus (AF), the frontal aslant tract (FAT), the Cortico-spinal tract (CST), the cingulum (Ci) and uncinate fasciculus (UF), and Optic radiation (OR) were reconstructed to display white matter infiltration frequency. Spatial normalization of segmented tumors, Brain-Grid analysis, and white matter reconstruction were performed using the built-in software of DSI studio (DSI Studio, http://dsi-studio.labsolver.org/download-images). Visual quality assurance was performed on all image normalizations.

**Figure 2:**
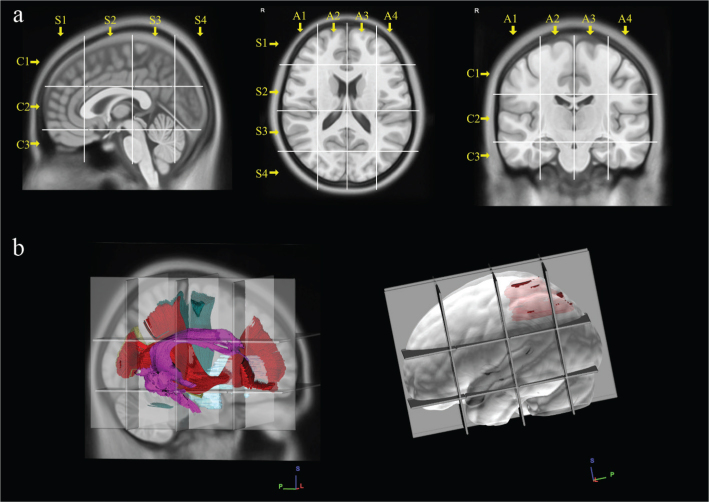
The picture shows the construction and use of Brain-Grid system into MNI space. (a) Three sagittal lines cross the anterior insular point (the most anterior landmark of the insular sulcus; MNI: Y-28, Talairach: Y-25 coordinates), the posterior insular point (MNI: Y-23; Talairach: Y-24 coordinates), and the temporo-occipital junction (between the posterior portion of the fusiform gyrus and the inferior occipital sulcus more basally on the axial plane; MNI: Y-68; Talairach: Y-66). These lines segment the whole brain into four grid voxels labeled with the first coordinate S (from sagittal line). The S1 voxel is the pre-insular/prefrontal portion of both hemispheres. The S2 is enclosed within the anterior insular point and posterior insular point (landmark for the second sagittal line). The S3 includes the retro-insular region and the parietal lobe, and the S4 includes primarily the occipital lobe and the border with the parieto-occipital sulcus. On the coronal plane, two parallel lines cross the inferior insular point (the lowest limit of the insular sulcus), the floor of the third ventricle, and the mammillary bodies (MNI: X-0, Y-5, Z-13; Talairach: X-0, Y-7, Z-7), while the second line passes through the cistern/space between the Cingular gyrus and the callosal body in the midline (MNI:X-0, Y-5, Z33; Talairach: X-0, Y-4, Z-31). Three voxels are created and named after the coordinate C (from coronal plane) with C1, which is the supra callosal, C2 between the corpus callosum and the mammillary bodies, and C3, which includes the region of temporal lobe, occipital lobes, and brainstem/cerebellum under the mammillary bodies. On the axial slices, the middle frontal sulcus bilaterally (right line, MNI: X-33; Talairach: X-32. left line, MNI:X-33; Talairach: X-32), and the midline (MNI: X-0; Talairach: X-0) are chosen as three landmarks for three parallel lines. In this way, four longitudinal segments are created, termed A1 to A4, from the right lateral side to the left lateral side. A total of 48 Brain-Grid voxels are created by the intersection of three sagittal lines, two coronal lines, and three axial lines. (b) Three-dimensional (3D) reconstructions of the tumor volume (in light blue) and crucial white matter anatomy (Arcuate fasciculus, magenta; Inferior occipito-frontal fasciculus, dark red; CST, green and optic radiation on the right side) analyzed with in Brain-Grid. On the right side, the 3D reconstruction of the left side hemisphere with a 3D reconstruction of tumor volume (in red) and the Brain-Grid system used for quantitative calculation of the number of infiltrated Brain-Grid voxels.

The Brain-Grid was reconstructed into MNI space intersecting standardized longitudinal lines on the axial, sagittal, and coronal planes on a T1-weighted average brain in the MNI space (see Figure 2a). As previously described, superficial and consistent cortical/gyral anatomical landmarks were originally chosen based on their bilateral symmetry and their relationship with subcortical white matter architecture. Depending on the planes (axial, coronal, or sagittal), several but constant anatomical landmarks were selected as previously described for this technique. On the coronal plane, the sulcus between cingulum cortex and corpus callosum, the mammillary bodies, and superior temporal sulcus were chosen. On the axial slices, the middle frontal sulcus on both sides and the midline are chosen. On the sagittal slices, the parieto-occipital sulcus, the temporal-occipital junction, and the anterior and posterior insular points were identified and used to originally reconstruct the overlay lines (25). In our study, the lines were uploaded into MNI space as pre-saved 3D objects. The Brain-Grid was constructed by three axial lines, two coronal lines, and three sagittal lines, whereas the intersection of these lines creates 48 grid voxels. Each voxel could be identified using simple nomenclature with radiological orientation. In the axial (A) plane, voxels are labelled 1–4, right to left direction. In the coronal (C) plane, voxels are labeled 1–3, cranio-caudal direction. In the sagittal (S), voxels are labeled 1–4, anterior-posterior direction (25).

As the reconstruction of the Brain-Grid voxels into MNI space was standardized, the size and position of each voxel was the same for all the subjects, as previously described (25).

Age, gender, and epileptic onset were included as clinical variables. Volume, side of lesion, well-defined, or diffuse tumor borders were included as radiological/topographical variables. The number of Brain-Grid voxels was analyzed as infiltration variables (Figure 2b) as previously described by our group (28). Initial surgical indication (biopsy or resection), extent of resection, and reoperation were included as surgical variables.

### Statistical analysis

For descriptive analysis, mean values and standard deviation (SD) were calculated for age, volume, extent of resection, and survival from diagnosis (years). Median and interquartile range (IQR) were calculated for the number of Brain-Grid voxels. Total values and percentages were calculated for gender, epileptic onset, side of invasion, radiological tumor borders, initial surgical choice, and reoperation. A Mann‑Whitney U-test for independent samples was used for group comparison between IDHm and IDHwt for numerical variables. Pearson’s Chi-square test was used for categorical (or binary) variables. For the number of Brain-Grid voxels and tumor volume, an optimal cut-off choice in two groups was made according to receiver-operating characteristic (ROC) curves. We calculated the test of equality of survival distribution using log rank (Mantel-Cox) analysis association between survival and each variable in IDHm and IDHwt patients. OS was calculated from dates of biopsy or surgical intervention to death or last follow-up. We examined all variables in the proportional hazard analysis (Cox model) to identify independent predictors of survival. A multivariate forward step-wise proportional hazard modeling was performed to assess the relative and independent prognostic capacity of each parameter. All statistical analyses were performed at a significance level of *P* < 0.05 and a confidence interval of 95%, using the statistical package SPSS 29.0 (SPSS, Inc., Chicago, IL).

## Results

Eighty-six patients received a histological diagnosis of WHO 2 astrocytoma between 2005 and 2015. Sixty-two patients (>18 years) were then included in this study based on the quality of radiological investigations, absence of contrast enhancement. Molecular confirmed status, according to the WHO 2021 classification criteria, was available in 40/62 astrocytomas, revealing 26 IDHm profile and 14 IDHwt. In the remaining 21 cases, the IDH status was unavailable and therefore excluded from further survival analysis for the current study. A detailed description of the genetic and molecular status of the IDHwt cohort is displayed in Supplementary material 1. Clinical and radiological information for the analyzed cases is summarized in Table 1.

**Table 1:** Summary of the clinical, topographical, radiological, and surgical data of the population divided in two groups according to the IDH status.

Clinical/radiological factors	IDHm	IDHwt	*P*
Number of patients	26	14	
Age *Mean (SD)*	39 (14)	42 (18)	0.740
Gender *- m (%)/ f(%)*	17 (65) / 9 (35)	4 (28) / 10 (72)	0.026*
Epilepsy - *y (%)/n (%)*	21 (80) / 5 (20)	11 (78) / 3 (22)	0.629
Side of invasion *‑ left (%)/right (%)/bilateral (%)*	16 (62) / 9 (34) / 1 (4)	8 (57) / 5 (35) / 1 (8)	0.922
Radiological border *‑ Defined (%)/Diffuse (%)*	11 (43) / 15 (57)	4 (29) / 10 (71)	0.079
Volume in ml *‑ Mean (SD)*	55.4 (52.3)	67.4 (55)	0.740
Brain-Grid Voxels *‑ Median (IQR)*	4.5 (2.75–8.25)	8.5 (5.7–10.2)	0.020*
Surgical Indication *‑ resection (%)/biopsy (%)*	21 (81) / 5 (19)	7 (50) / 7 (50)	0.043*
Resection grade *‑ mean % (SD)*	79.2 (20.7)	73.1 (21.2)	0.385
Reoperation *‑ y (%)/n (%)*	14 (54) / 14 (46)	6 (43) / 8 (57)	0.507
Deceased *‑ y (%)/n (%)*	15(57) / 11 (43)	11(78) / 3 (22)	0.187
Survival from diagnosis *‑ years mean (SD)*	8.8 (3.3)	5.8 (3.5)	0.037*

A Mann‑Whitney U-test for independent samples was used for group comparison between IDHm and IDHwt for numerical variables. Pearson’s Chi-square test was used for categorical variables

(*statistically significant for *P* < 0.05).

M: male subjects; F: female subjects; y: yes; n: no; WM: white matter; BG: Brain-Grid system. IQR: Interquartile range.

**Figure 3:**
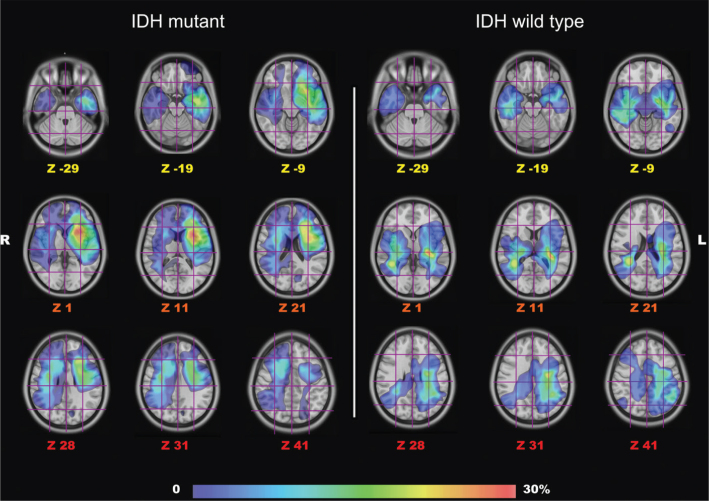
The picture shows the gradient maps reconstructed from the fusion of each tumor region within the MNI space. Each tumor was manually segmented and saved as binary images. Statistical maps of tumor location frequency were obtained by computing for each voxel the cumulative number of observed lesions/tumors. The frequency of tumor location for the IDHm group (total of 26 tumors) is shown on the left side. The distribution of IDHwt tumors (14 tumors) is shown on the right side. On each axial slice localized with Z coordinates (MNI space), the Brain-Grid System is displayed as overlay and used as a reference for BG voxel count. The frequency of tumor location for the two populations is color graded (0–30% in the gradient scale) according to the rate of voxel infiltration. R: right side; L: Left side.

The mean follow-up time was 7.8 years (range 0.6–15). Analysis of the clinical variables revealed a significative higher percentage of male patients in the IDHm group and female predominance in the IDHwt group (*P* < 0.05). The two groups displayed the same age distribution and frequency of epileptic onset. IDHm astrocytomas displayed a significant lower number of Brain-Grid voxels compared with IDHwt group (*P* < 0.05). In all the cases, the tumor contour line was considered either well defined or diffuse; no one patient displayed both features at the same time. No significative differences were detected regarding the other radiological variables between the two groups. The fronto-temporo-insular subcortical area on the left side was the most frequent location (30%) in IDHm cases while the posterior insular region and the peritrigonal region on both sides were the most commonly involved in IDHwt patients (Figure 3).

**Figure 4:**
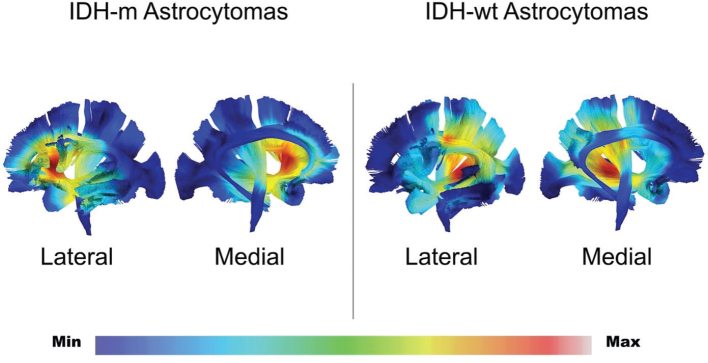
The picture shows the gradient maps reconstructed from the fusion of each tumor region within the MNI space in IDHm astrocytomas and IDHwt astrocytomas. The gradient maps were used as overlay for the reconstructed white matter bundles on the left hemispheres in both lateral and medial views. The left IFOF, AF, FAT, UF, CST, and Ci have been reconstructed to display infiltration frequency of each pathway. The frequency of tumor location for the two populations is color graded (0–30% in the gradient scale according to the number of cases) according to the rate of voxel infiltration as previously described.

The white matter analysis in the two populations showed preferential infiltration of the anterior-dorsal IFOF within the external capsule and the lower portion of the FAT in IDHm cases while the posterior portion of IFOF, AF, and ventral part of the CST in IDHwt patients (Figure 4).

**Figure 5:**
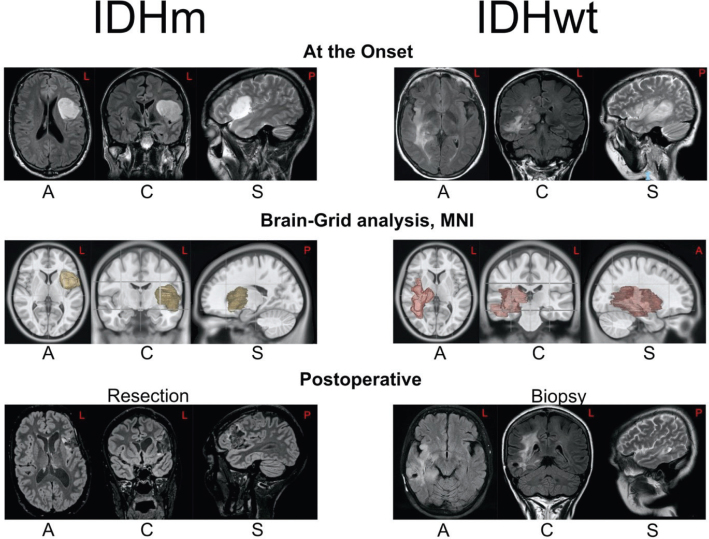
The picture shows two illustrative cases to better describe the radiological and clinical features of each group. On the left side, a case of IDHm astrocytomas is displayed. The tumor was detected after epileptic seizure (age <40). The patient underwent an MRI showing on axial (A) and coronal (C), flair and sagittal (S), T2-weighted sequences a suspected low-grade glioma harboring the left fronto-opercular area and anterior-dorsal insula. The MRI showed sharp borders, and at the retrospective Brain-Grid analysis, the tumor invaded three voxels with a volume of 38 ml. Because of the eloquent position and the positive findings of slight speech impairment, the patient was operated with awake surgery until the functional limit was detected. The postoperative images show a minimal tumor remnant at the level of insula. The patient underwent a postoperative radiotherapy as first choice, and during the follow-up, a chemotherapy treatment was started, with at the last follow-up an OS of 14 years from the radiological diagnosis. On the right side, an illustrative case from the IDHwt group. This patient displayed an onset with olfactory hallucination, paresthesias, and dysarthria (age > 60). The radiological investigation showed a suspected low-grade glioma invading the peritrigonal area on the right side with involvement of insular cortex, basal ganglia, and mostly white matter into the sagittal stratum of Sachs. MRI investigation showing on axial, coronal (FLAIR), and sagittal (T2W) showed diffuse, irregular borders, and at the Brain-Grid analysis, the tumor invaded a total of 10 voxels with a volume of 58.5 ml. Considered the position mostly subcortical, the extension, and the age the patient underwent a diagnostic biopsy showing IDHwt astrocytoma. The patient underwent postoperative radiotherapy with an OS of 7 years.

The initial choice of a biopsy was significantly higher in IDHwt astrocytomas while there was no difference regarding the other surgical variables (extent of resection, reoperation). Two illustrative cases are displayed in Figure 5 to better describe the typical patient features for each group.

Univariate analysis was performed with the Kaplan‑Meier method, with comparisons using the log-rank test. Radiological tumor borders, side of invasion, preoperative tumor volume, number of BG-voxels, initial surgical choice, resection grade, and eventual reoperation/multistep surgery as explanatory variables (Table 2). In IDHwt patients, the crucial number of 7 or more Brain-Grid-voxels was associated with a significative shorter OS (χ^2^ = 4.661; *P* < 0.05). Multivariate proportional hazard modeling was performed for each outcome measure using the following parameters: gender, radiological border (diffuse), optimal volume cut-off, BG-voxels cut-off, initial surgical choice, and reoperation/multi-step surgery. Brain-Grid voxels (>7) were selected by the equation as independent prognostic factors in IDHwt astrocytomas able to predict a shorter overall survival. In IDHm astrocytomas even if selected by the equation, it did not reach a significative *P*-value (Table 2).

**Table 2:** The upper part of the table shows the test of equality of survival distribution using log rank (Mantel-Cox) analysis with OS as outcome variable and clinical, radiological, and surgical variables in IDHm and IDHwt astrocytoma subgroups as test variables.

Analyzed variables	IDHm			IDHwt		

Univariate analysis	*p*	χ^2^		*P*	χ^2^	
Gender - m	0.918	0.011		0.236	1.405	
Radiological border - diffuse	0.301	1.070		0.975	0.001	
Volume - > op C-off	0.179	1.809		0.158	1.997	
Brain-Grid Voxels - > op C-off	0.125	2.358		0.038*	4.314	
Surgical indication - resection	0.890	0.019		0.294	1.103	
Reoperation/multistep surgery - y/n	0.255	1.298		0.510	0.434	

Multivariate analysis	*p*	HR	95% CI	*P*	HR	95% CI

Brain-Grid Voxels - > op C-off	0.197	0.485	0.162–1.456	0.023*	2.995	1.161–7.729

The lower part of the table shows the multivariate Cox regression analysis with proportional hazards modeling (forward conditional), which was performed to assess the relative and independent prognostic capacity of each parameter (gender, BG voxels >5 in IDHm, and >7 in IDHwt, surgical indication was chosen as variables). Only BG-voxels > 7 were selected by the equation as independent prognostic factors.

(*statistically significant for *P* < 0.05).

M: male; f: female; y: yes; n: no; OS: Overall survival.HR: Hazard Risk; O c-off: Optimal cut-off defined by ROC curves.

## Discussion

This retrospective study showed three main results. First, a difference between IDHm and IDHwt patients was observed in the topographic distribution of the tumors. Observing the gradient maps, the hot spots for the infiltration frequency were subcortical at the level of insula and periventricular areas (Figure 3). IDHm showed a significant preferential left anterior insular location, while IDHwt displayed a more prominent posterior insular and peritrigonal location. This seems in line with the previous contribution showing the value of gradient map analyses in detecting differences in the tumor topography (14, 15) and thus differences in preferential location among different types of DLGG (17). The subsequent analysis of white matter involvement displayed higher infiltration frequency of the posterior IFOF and CST for the IDHwt tumors compared with anterior dorsal IFOF and the FAT in IDHm astrocytomas. The topographical and white matter infiltration analysis in this article confirms the difficulties in the surgical management of these lesions harboring the so-called ‘minimal common brain’ (14). However, the IDHwt group displayed a more complex location and infiltration of eloquent bundles. It seems not surprising that 50% of IDHwt patients received a diagnostic biopsy instead of a surgical cytoreduction as the initial surgical management. This higher rate of biopsies may reflect the risk of damaging eloquent systems such as CST but also the risk of leaving more residual tumor considering the extensive subcortical extension (periventricular/posterior insular) as showed by illustrative cases in Figure 4. It is also mandatory to consider that this study reflects the decade 2005–2015, where the use of MRI tractography, the use of awake surgery, and neuropsychological testing were not fully established in our center. However, the favorable impact on overall survival of surgical resection compared to biopsy is nowadays commonly accepted and cannot be neglected (4, 19, 29). We agree with the paradigmatic shift compared with the last decade, toward a more prominent role of surgical resection according to functional boundaries to increase resection rate respecting the white matter within the minimal common brain (14, 15, 30–32). We have then improved perioperative planning (including DTI tractography), our surgical strategy with direct cortical-subcortical mapping, and monitoring of high cognitive functions. We have, indeed, re-operated over 40% of the patients in both groups during the follow-up time to increase the extent of resection or due to radiological progress according to functional boundaries.

Second, the number of infiltrated BG-voxels was significantly different between the two groups. IDHwt astrocytomas displayed a more prominent subcortical infiltration than IDHm astrocytomas. This information cannot be extrapolated from classical topographical/radiological classification systems (4, 21, 23, 24). These systems are based on ‘local’ neuro-radiological anatomic features; hence it is common practice to describe a glioma based on the nomenclature of major lobes invaded despite the subcortical extension (4, 21, 23, 24). The tumor extension has almost exclusively been described by volume measurement in the recent literature (29, 33–35). Our results show that there was no significant difference in terms of volume between the two populations (Tables 1 and 2). We classified low-grade gliomas using a standardized radiological tool of intersected lines according to anatomical landmarks: the Brain-Grid. (25). The main advantage of this technique is the possibility to integrate topographical information, radiological features, and tumor volume in low-grade gliomas including quantitative and qualitative information on infiltrated white matter. Despite the tumor volume and the number of BG-voxels intuitively correlated, the BG system does reflect only the size of the tumor, which can be confined to a single anatomical region, i.e. well-defined tumor of the frontal pole. On the contrary, tumor with similar volume but located in deep regions such as periventricular areas showed a more prominent subcortical extension with a higher number of infiltrated Brain-Grid voxels (25).

The third result is that the difference in IDH status reflected different outcomes at the long follow-up time and despite the multi-step surgeries in both groups. This is in agreement with other authors describing a clear difference in OS linked to IDH status (7, 8, 36, 37). However, when we looked at possible factors affecting the OS in the two groups, the only variable affecting the OS was the number of Brain-Grid voxels and only in the IDHwt group. We believe that the number of BG-voxels may reflect the invasiveness of gliomas (17, 25). Our results showed that the invasion of more than 7 BG-voxels in IDHwt astrocytomas at the radiological diagnosis may predict the outcome independently of tumor volume or resection grade. The different in the white matter invasion seems particularly important with regard to new proposed models of individualized management algorithms (9, 22, 38). The prominent infiltration of white matter by the tumor is considered a negative prognostic factor reflecting high invasive feature and affecting the neuroplasticity potential of the host brain. In such cases, a surgical resection needs to be carefully evaluated with a prediction of residual tumor volume. Therefore, in selected cases, a diagnostic biopsy may also be considered as an initial surgical choice to reduce the possible surgical complication in light of a worse functional and oncological prognosis with a lower extent of resection (9, 22, 34, 38–40). In our study, a prominent white matter infiltration was described by a higher number of BG voxels, which affected the OS in IDHwt astrocytomas. This observational tool may be helpful in interpreting the preoperative features of gliomas and may be used to quantify the prominent infiltration of white matter bundles. New developments in pharmaceutical treatment (preoperative chemotherapy (41, 42) or IDH inhibitor (43, 44)) of DLGGs may play a role in the future as a possible preoperative help in reducing the white matter infiltration and leading to a better resection rate.

## Limitations

Our study has some limitations. A possible criticism to this study may arise from the choice to include only confirmed astrocytomas. As these lesions are considered among the most challenging by neurosurgeons and display tendency to high infiltrative behavior, we believe that they represented a perfect indicator of the relationship between invasiveness, preferential locations, and surgical challenge.

Patient data were included from two different MR scanners at different field strengths, which may introduce different delineation errors during tumor segmentation. Only a few patients were enrolled in this study during the period 2005–2007 when a 1.5T camera was used, and the images based on FLAIR sequences were analyzed for quality before the inclusion in this study. Moreover, tumors were relatively large; hence, any errors introduced during segmentation or normalization to standard space are considered minor. Therefore, we do not believe that the difference of cameras may have an impact on the actual results.

The sample size is small, and this may affect the impact of our conclusions. However, our aim was to identify possible associations between IDH status and anatomical, clinical, radiological, and surgical features during a long follow-up time. We therefore believe that, despite the small sample size, our cohort may represent a clean comparison between two different biological entities that display a similar clinical and radiological onset.

Another important limitation is that the outcome of the two populations may be affected by other factors not analyzed in this study and therefore should be carefully interpreted. IDHwt astrocytomas are nowadays treated differently also based on other molecular or genetic features. Due to the retrospective nature of this study and the period of onset between 2005 and 2015, patients in both groups have been treated according to different protocols and WHO classification systems. A more extensive genetic and molecular assessment of the IDHwt group is provided as supplementary material but not analyzed in this study. Differences among the subjects in regard of the analyses performed and the small sample size make us unable to clearly define other genetic or molecular features linked to clinical and radiological aspects. As the main aim of the study was to identify possible predictors of IDH status at the preoperative onset, the OS in our cohort should be interpreted as secondary result, which agrees with the reported literature.

Finally, the choice of using normalized MNI space may raise some criticism. We adopted the Brain-Grid system because it can work in both patient specific space and MNI space. The real advantage with the normalization process is the interobserver reproducibility and the possibility to collect a gradient map of the frequent tumor location analyzing even the difference between selected populations (Figure 3). However, because of the image distortion, the volume and radiological border features were calculated into patient-specific space. The number of Brain-Grid voxels and the infiltration of white matter pathways were recorded into MNI space because of the quantitative consistency in the voxel counts demonstrated by previous publications (25).

## Conclusions

We found that IDHm astrocytomas are less invasive (according to BG classification system) and preferentially localized in the anterior sub-insular region/IFOF on the left side. IDHwt tumors are more invasive and often localized in the posterior insular region or peritrigonal white matter (IFOF/CST) on the left side. The number of BG-voxels, possibly reflecting differences in tumor invasiveness, affected the OS in IDHwt astrocytomas.

Prospective multicentric studies and a systematic use of more accurate observational tools, such as gradient maps and the Brain-Grid analysis, would be able to better define differences among tumor subtypes and define the importance of tumor invasiveness in suspected DLGGs.

## Supplementary Material



## Data Availability

The data that support the findings of this study are available on request from the corresponding author. The data are not publicly available due to privacy or ethical restrictions.

## Supplementary materials

A detailed description of the genetic and molecular status of the IDHwt cohort is provided to better understand differences in the outcome. The supplementary materials can be accessed here.
