# Supplementary material for: Anatomical and subcortical invasiveness in diffuse low-grade astrocytomas differ between IDH status and provide prognostic information
Source: Ups J Med Sci. 2024 Sep 3;129:10.48101/ujms.v129.10799. doi: 10.48101/ujms.v129.10799 (PMC11375500; doi:10.48101/ujms.v129.10799)
Supplement: Supplementary file 1 [file UJMS-129-10799-s1.pdf]

Gender	First Symptom	Side	Initial surgical choice Resection/ Biopsy	Survival from radiological diagnosis	Reoperation	IDH status	1p19q	KI-67	EGFR	TERT	MGMT	GFAP	P53	ATRX
F	Dysphasia, hemiplegia	L	B	3	Y	wt	-	3	+	N/A	2	+	10	N/A
F	Vertigo, Seizures	L	R	2	N	wt	-	1	+	N/A	N/A	+	1	N/A
F	Smell Hallucination, paraesthesias	R	B	7	N	wt	-	<1	N/A	N/A	9	+	0	+
M	Seizures	R	R	3	Y	wt	-	8-10	+	N/A	N/A	+	0	+
F	Weakness right arm and face	L	R	4	N	wt	-	1-5	W	N/A	N/A	N/A	15	M
F	Seizures	R	B	3	N	wt	-	7-8	W	N/A	N/A	+	0	+
M	Seizures	L	B	10	N	wt	-	4-7	+	N/A	N/A	+	0	N/A
F	Seizures	R	R	7	Y	wt	N/A	8-10	+	N/A	N/A	+	0	N/A
F	Paraesthesias vertigo, Seizures	L	R	6	y	wt	-	2-5	+	N/A	N/A	+	50	+
M	Seizures	L	B	12	N	wt	-	1-5	+	N/A	N/A	+	0	+
M	Headache, seizures	L	B	4	N	wt	-	5-6	+	N/A	N/A	+	0	+
F	Seizures	R	R	7	Y	wt	-	5	+	N/A	N/A	+	1	+
F	Seizures	B	B	1	N	wt	-	<1	+	N/A	N/A	+	0	N/A
F	Seizures	L	R	4	Y	wt	-	10	-	N/A	5	+	0	N/A

Supplementary material 1. The table summarizes the clinical and genetic/molecular results of the IDHwt group. On the left part of the table are displayed gender (male, M or female, F) Clinical onset, side of tumour presence (left, L, right, R, or bilateral, B), the first surgical choice (biopsy , B or resection, R), survival from radiological diagnosis in years. On the right part of the table the genetic molecular results are shown. IDH status reflected in all the 14 cases absence of the most important mutations therefore wild type (wt); Codeletion of 1p19q chromosomes was negative in all the cases except one where was not available (N/A). Ki67 is displayed in numbers reflecting observed percentage; Epidermal growth factor receptor (EGFR) is displayed with plus

sign if positive (+), minus sign if negative (-), the letter W indicates that the expression was weak and in one case was not available (N/A). Telomerase reverse transcriptase (TERT) promotor mutation was not available in our cohort on suspected low-grade gliomas reflecting the retrospective nature of this study . O6-methylguanine-DNA methyl-transferase (MGMT) promotor was available in only few patients and displayed in numbers reflecting the percentage. Glial fibrillary acidic protein (GFAP) is displayed with plus sign if expressed; P53 gene mutation displayed in numbers reflecting the observed percentage; Alpha thalassemia/mental retardation syndrome X-linked mutation (ATRX) is displayed with plus sign if positive and with letter M if mutated.
